# Some Practical Points in the Diagnosis and Treatment of Acute and Chronic Aural Suppuration and Their Sequellae

**Published:** 1913-03

**Authors:** James F. McKernon

**Affiliations:** New York City


### Some Practical Points in the Diagnosis and Treatment of Acute and Chronic Aural Suppuration and Their Sequellae

By JAMES F. McKERNON, M. D.
New York City

IN presenting this paper for your consideration this evening, I have been requested by your President to deal with the subject more from the standpoint of the general practitioner than from that of the specialist. In doing so I must crave your indulgence if I touch upon many phases of aural suppuration that are more or less familiar to you.

Frequently the general practitioner is called in to see a young child, with the history that the child has been restless, irritable and unable to sleep, with some accompanying temperature. This may have followed a cold, sore throat, tonsilitis or it may be independent of any previous general or local condition. Here a physical examination of the child is not complete unless it includes an examination of the ears, and when such an inspection is made one or both ears is frequently found involved in an acute inflammatory process which readily explains the cause of the little patient's discomfort and temperature.

It is a popular fallacy among many, that when an ear first becomes the seat of an acute inflammation, that the first symptom the child will complain of is pain, referred to the ear. Occasionally they will do so, but the majority of very young children will give no such evidence until several hours or days have elapsed, or until a discharge is noticed on the pillow, when the general systemic symptoms usually subside, or the aural condition is recognized by an early otoscopic examination.

Another phase of aural suppuration which is misleading, is to have an older child complain of sporadic attacks of earache, with an irregular temperature curve. Frequent physical examination eliminates the condition as being caused by one of the many systemic diseases usually looked for in childhood. The ears are finally subjected to an examination because of the occasional attacks of pain formerly complained of by the child.

In examining these cases, it is infrequent that a hasty aural inspection with an insufficient light is made, sometimes by one not well versed in otoscopic examinations, or as has many times been the case, the examination has been made hurriedly by the aurist, and the only thing noticed about the drum membrane as deviating from normal was that it was somewhat prominent, with absence of lustre, and looking, to use a common expression, "dead white." The examiner seeing such a condition, free from any acute congestion, usually gives some simple instructions for the care of the child and takes his departure, saying there is no involvement of the ears.

Uusually in a few hours afterward he is sent for, or telephoned that the child has a discharge from the ear. He visits the patient as soon as possible to find a rather profuse discharge from the ear that he had pronounced negative but a few hours before, and naturally thinks that the process must have been an unusually rapid one. Had he brought to his aid at the first examination a cotton-tipped probe, and gently brushed the surface of the drum membrane from above downward, he would have removed the dead-white, opaque looking surface, which is composed of exfoliated epithelium, due to pressure of fluid behind the drum membrane, and found a bulging, swoolen and reddened drum, which should have been incised at once.

It is my firm belief that if every case of measles, diphtheria, scarlet fever, acute tonsilitis, pneumonia and typhoid fever, as well as many other of the acute diseases, were subjected to frequent otoscopic examinations during the course of the disease, that the best interests of the patients would be conserved, for many times during the course of one or more of these diseases such an examination would reveal the cause of an unexplained temperature.

For such frequent examination it is not necessary that an aurist be called. The general practitioner should be able to make these inspections and determine between the normal and pathological looking drum membrane and middle ear. It is in many of these systemic diseases that a slow, non-painful, insidious involvement of the ear takes place.

Another dangerous phase of acute aural suppuration in infants and young children is the continuation of an irregular temperature curve several days after the onset of an acute middle-ear involvement.

Take, for instance, a case where the drum membrane has ruptured spontaneously, or has been incised late in the course of an acute aural suppuration, and drainage of the middle-ear is complete, but in spite of free drainage and active antiphlogistic measures, an irregular temperature persists for several days, 99° F. to 100° F. in the morning, 100½° F. to 101° F. in the afternoon, or normal in the morning with a rise to 102° F. to 103° F. in the evening with a reversion to normal the next morning. Physical inspection of the ear shows the canal filled with pus, either creamy, or white and stringy, depending upon the characteristic bacterial infection producing the inflammation, and when the canal is cleansed of all secretion, there is noticed only a moderate amount of fulness of the posterior canal wall. The drum membrane is not bulging unduly, but simply showing an oedematus and thickened condition, in fact few, if any, of the classical canal and middle ear signs of a mastoid involvement are present, yet if we watch such a middle ear and canal for from three to five minutes we will find the canal quickly filled to the meatus with the same characteristic secretion that was removed but a few moments before. Can anyone think for a moment that a cavity as small as that of the middle ear can hold such an amount of secretion?

The suppurative process has extended to the mastoid bone and the large amount of pus accumulating in the canal is passing through the middle ear from the suppurating process already existing in the mastoid structure. And this, with no external physical signs of bone involvement, such as tenderness upon pressure over the antrum or tip of the mastoid, or swelling or oedema over this structure.

Later when swelling and oedema does take place it is because the natural drainage to the mastoid antrum, "the aditus," has become blocked with granulation tissue, and there is a still more pronounced extension of the general mastoid structure; or a cortical perforation has taken place, allowing the pus to escape from the bony cavity upward beneath the periosteum, producing a sub-periosteal abscess.

It is practically impossible in these infants and younger children to elicit the symptom of pain upon pressure over the mastoid area. They will cry out and complain quite as much if pressure be exerted on the opposite side, or on top of the head as on the suspected side, and he who waits for these children to show a swelling over the mastoid, or a sub-periosteal accumulation, before advising an operation on the mastoid, is inviting one or more of the intra-cranial complications, that unfortunately follow in the wake of these neglected and delayed cases.

It was formerly said that when these sub-periosteal swellings occurred, the only treatment that was necessary was a free incision through the soft parts and periosteum to the bone—the so-called Wilde's incision.

Believing that such treatment was unsafe, I was enabled several years ago to prove, at least to myself, the danger of such a procedure.

In 166 cases of sub-periosteal abscess complicating acute and chronic aural suppuration in children, ranging in age from three to ten years, the mastoid was opened, and in 165 of the cases pus was found in the entrum and throughout the mastoid structure generally. In the single case where it was not present, the antrum was filled with granulation tissue, and the general osseous structure found red and softened. While some of these cases might have been relieved by the simple external incision, the majority would not, but would have been left with the legacy of a running ear for the remainder of their lives, with impaired audition, and with favorable conditions for the subsequent development of one or more of the various intra-cranial complications, so frequently seen in neglected cases of this type.

In adult cases the otologist is frequently asked, what symptoms or physical signs are present that enable him to make a positive diagnosis of mastoid involvement?

They are the presence of a discharge from the ear of the affected side, or the history of such a discharge, partial or complete, prolapse of the posterior-superior canal wall, shutting off from view a portion of a bulging drum membrane, especially that part known as the posterior-superior quadrant, so that when we are examining the ear with the speculum this part looks nearer to us than the anterior-inferior quadrant, showing a decided narrowing of the lumen of the canal in its deeper third, tenderness upon pressure over the mastoid antrum, tip, or the exit of the emissary vein, occasionally tenderness upon pressure over the middle zigomatic root. In some cases the tenderness is pronounced only over the antrum and tip. If the case be one prolonged or delayed, then tenderness may be exhibited over any point where pressure is exerted.

Temperature depends entirely upon the stage at which the disease is seen. During the development of pus there is always a temperature. When pus has once formed in the mastoid cavity, there is usually a subsidence of all temperature, until complications begin to arise. This absence of temperature is often misleading, and is frequently the cause of much unnecessary delay and waiting. Here the physical signs of the canal and middle-ear, with surface tenderness at the points mentioned, is sufficient for a positive diagnosis. Occasionally we come in contact with cases, both in young children and adults, that do not exhibit any tenderness whatever upon pressure. This lack of sensitiveness is usually due to a very thick external cortex. Here the diagnosis is made from the canal and middle-ear charges alone.

At times we have only the history of a discharge having been present some two or three months previously, and no tenderness exhibited upon pressure. Here the diagnosis is made upon the canal changes already spoken of.

Audition is invariably diminished following the development of a suppuration in the mastoid, and varies with the extension of the disease. If of long standing, several weeks in duration, it steadily becomes worse, due no doubt to the excessive inflammatory changes that have taken place in the middle-ear cavity. Diffuse circumscribed external otitis, or ferunculosis of the external auditory canal, is sometimes mistaken for mastoid involvement, especially if it be well developed and has lasted sufficiently long to cause a well marked oedema behind the auricle and over the mastoid process. Physical examination of the external auditory meatus reveals the lumen of the cartilaginous canal as well as the meatus encroached upon to a greater or less degree by a swelling in one or more of the canal walls. This swelling is excessively tender upon pressure. Pressure with the finger in front of the tragus, or underneath the lobe of the ear upward, or in the post aural fold toward the auricle all produce most exquisite pain. Movement of the auricle between the thumb and finger will produce a like effect.

Pressure over the oedematus mastoid swelling does not as a rule cause pain. Should the swelling be excessive and the pressure causing pain in this region, it is due to the swollen soft parts and not to the bone surface below. The treatment is free incision of the swollen area in the meatus and canal. The taking of a culture from the evacuated discharge, and then covering the entire ear and mastoid area with a plentiful supply of plain sterile gauze, soaked in a hot normal salt solution, covered with rubber tissue, and applying a bandage.

From the culture taken an autogenous vaccine is to be made and injected, to hasten resolution and prevent the formation of other circumscribed areas of infection. The infection is usually that of the Staphylococcus and the initial dose of the vaccine should be for the adult 500,000,000 bacteria, with subsequent doses, larger or smaller, every three or four days, depending upon the rapidity of resolution in the affected tissue.

Probably there is no aural disease that the general practitioner as well as the otologist is more frequently consulted about than that of a "running ear," and frequently in the past the patient has been told by his medical adviser to let it alone, and that nature would effect a cure. In some of the cases where there is absence of necrosis of the middle-ear structures and adjacent parts, nature will do much in effecting a cure. But, if necrosis of these structures be present, then their continuance means a menace to the well being and future health of the patient. In every case of a discharging ear coming under our observation, whether it be acute, sub-acute or chonic, a most careful examination should be made. First to discover the cause of the discharge, whether it be due to insfficient drainage alone, or if chronic, whether necrosis of the ossicular chain or adjacent structures exist. If drainage be insufficient, this should be corrected by a free incision of the drum membrane, followed by antiseptic and stimulating treatment on conservative lines, hoping thereby to bring about resolution and cure the suppuration. Formerly we were in the habit of performing an ossiculectomy in nearly every case of chronic otorrhoea that persisted after conservative treatment, independent of whether the disease was confined to the ossicular chain or to the deeper structures, with the result that only a very small percentage of our cases were cured, namely, those in which the disease was confined to the ossicles alone.

The vast majority of our cases operated upon in this way continued to discharge, because it was impossible to reach the seat of the trouble by an operation performed through the external auditory canal, for when this was done areas of necrosis were left untouched in the attic, around the eustachian orifice, in the aditus, and in the mastoid bone itself. In many of these cases so operated upon a meningitis developed, due to the fact that there existed one or more perforations through the tegmen tympani, communicating with the dura above, the dura in this region having previously been the seat of a pachymeningitis which was nature's barrier for the time being, to any further progress of the suppuration in this direction.

Instrumental manipulation within the middle-ear during the ossiculectomy was sufficient to cause an irritation and re-infection of the dura which rapidly developed into a general and fatal purulent meningitis. Then again, in these cases of operation through the canal, it was not an infrequent occurrence, to injure the facial nerve by the instrumental manipulation that was carried on out of sight in the tympanic cavity, so that today if necrosis of the middle-ear be demonstrated by our examination, we invariably advise the patient to submit to the Stacke or radical operation, in order that the discharge may be cured, and that he may be placed on a safe basis from future intra-cranial developments.

Briefly stated, the causes demanding a radical operation of the middle-ear and structures adjacent thereto, are:

The presence of dead bone in the tympanic cavity. The presence of cholesteatomatous masses. Headache, *localized* or *general, on the affected side.* Vertigo of the intermittent type, associated with, at times, nausea and vomiting. Intermittent pain, or a dull, heavy, throbbing pain when drainage is obstructed. Unsteadiness of gait, and an intermittent or constant purulent discharge from the middle-ear, with a foul odor, indicative of necrotic bone.

Tinnitus is frequently present, and at times most distressing. Occasionally when the condition is of long standing there is considerable mental depression, almost a mild form of melancholia. Successive attacks of labyrinthine irritation during the course of a chronic suppuration in the middle-ear is an indication in itself sufficient for the performance of this operation.

I shall purposely omit mentioning the various functional tests for diseases of the labyrinth, as I wish this paper to appeal more to the general medical man than to the specialist.

Another class of cases frequently coming to the operating table for the radical operation, is that of children giving the history of a discharge from the middle-ear for several months as a sequellae of grippe, measles, scarlet fever and other intercurrent diseases. In many of these cases perisistent, conservative treatment has failed to bring about a cure. The adenoids and enlarged tonsils have been removed, hoping thereby, to lessen the mechanical irritation in the naso-pharynx, so that resolution will be established, but without success. If, instead of the radical operation being done on these children, the matoid were opened, the middle-ear drained posteriorily, and at the same time a free incision made in the drum membrane, the suppurative process would speedily cease, and what is of the utmost importance for the future of these young children, their hearing would be preserved, or vastly improved in the majority of cases; while if the radical operation had been done on these young children their future hearing would be very much diminished, if not entirely absent at the end of a year or two after such an operation.

During the last five years the writer has seen 106 cases of the above described type in children operated upon by the posterior method and all discharge cured except in ten cases. The audition in 84 cases was as perfect as that of the opposite side. Of the remaining twenty-two cases, the audition was improved in twelve. Six had to be operated upon again, the radical operation being done at the second sitting. The four remaining cases passed from under observation, and no trace of them was obtained for future reference.

The patient who contemplates having a radical operation performed usually asks four questions:

First: Will the discharge be cured? Second: Is it dangerous? Third: Will my face be paralyzed? Fourth: Will my hearing be improved?

The answer to the first question is: We expect that it will, although it may be necessary to do a second operation, in order to bring about a cure, depending upon the conditions that are found upon operation.

Second: We do not consider the operation a serious one, if no complications exist at the time of the operation, yet in every operation there is an element of danger.

Third: We do not expect injury to the facial nerve, although it is possible, owing to the fact that it may be exposed by extensive necrosis, and in removing such necrostic areas the nerve may be injured, or ot may have an anomalous distribution, and receive injury on this account.

Fourth: The hearing in the vast majority of cases will not be improved, but worse than before operation, and practically useless as far as hearing conversation. I have never seen but two cases of permanent improvement in audition following the complete radical operation, and during the earlier days in which we performed this operation, I have many times heard the patient state that had he or she known their hearing would have been as poor as it then was, that they would never have had the operation done.

Such statements as these, however, should have but little weight when we compare their diminished audition to the probability of a dangerous intra-cranial condition occurring, in cases where advanced necrosis is present, for I do not know of any operation that offers such safety to the patient as does this, when the indications are distinct and definite for its performance.

[Continued next month]

